# Impact of the COVID-19 Lockdown on Quality of Life in Pregnant Women

**DOI:** 10.3389/fpubh.2022.785383

**Published:** 2022-01-31

**Authors:** Jingjie Ma, Ating Wang, Hang Zhou

**Affiliations:** ^1^Department of Nutrition, Baoji Maternal and Child Health Hospital, Baoji, China; ^2^Clinical Medical College, Yangzhou University, Yangzhou, China; ^3^Department of Clinical Nutrition, Northern Jiangsu People's Hospital, Yangzhou, China

**Keywords:** IES, psychiatry, pregnant women, China, stress

## Abstract

**Background:**

The COVID-19 pandemic has been associated with significant impacts on mental health and well-being of populations worldwide. However, little is known about its significant impact on psychological aspects of vulnerable population groups such as pregnant women. Therefore, the aim of the study was to determine the psychological impact of the COVID-19 pandemic among pregnant women in mainland China.

**Methods:**

A cross-sectional survey was performed between July and August 2020 using a modified validated 40-item questionnaire which consisted of sociodemographics, attitude, lifestyle changes and the Impact of Event Scale (IES) toward COVID-19 using snowball and convenience samplings.

**Results:**

A total of 1,078 participants were included in the study. The mean age of participants was 29.4 ± 4.0 years. Overall, the mean IES of participants was 30.6 ± 12.8 (i.e., moderate-to-severe stressful impact) amidst the COVID-19 pandemic, with 63.9% of participants had an IES score ≥26. Despite increased family and social support, more than half of participants also reported increased feeling of being horrified, apprehensive and helpless.

**Conclusions:**

The COVID-19 pandemic has several psychological impacts on pregnant women. Therefore, based on these valuable data of pregnant women collected, we recommend that a thoughtful planning and time preparation by the government would definitely help to reduce the negative impacts caused by the COVID-19 pandemic and restore the quality of life among pregnant women. Further research is needed to identify vulnerable groups including pregnant women to better adapt and inform mental health interventions and policies by health authorities.

## Introduction

The emergence of coronavirus disease 2019 (COVID-19) caused by severe acute respiratory syndrome coronavirus 2 (SARS-CoV-2) firstly appeared in Wuhan, Hubei province, China, in late December 2019 (1). Within just a few months, it has already evolved into a pandemic. As of December 2021, more than 281 million confirmed cases and 5 million deaths have been reported (2). Since the COVID-19 is a highly infectious respiratory disease, isolation is needed to reduce the disease transmission of the COVID-19 pandemic, especially to the vulnerable groups. In addition, social isolation reduces the peak in COVID-19 cases (3).

Pregnant women are considered a vulnerable group for contracting the SARS-CoV-2 (4). In addition, they have increased risk of developing severe illness from COVID-19 when compared to non-pregnant individuals (5). The findings might be due to the physiologic changes in pregnancy including decreased lung capacity, increased heart rate, increased risk for developing thromboembolic disease. Therefore, higher adverse outcomes associated with COVID-19 including morbidity and mortality rate has been reported in pregnant women (6).

World Health Organization has recommended social isolation between individuals, especially in COVID-19 heavily-affected areas to reduce the mortality rate (7). Although social isolation reduces the transmission of SARS-CoV-2 between individuals, a significant reduction in social relationships would make them feel lonely and abandoned (8). Studies have reported that pregnant women are susceptible to stress and emotional instability (8–11). Therefore, it is suggested that social isolation can cause pregnant women to develop anxiety and depression. Therefore, the COVID-19 pandemic has posed huge and complicated challenges associated with mental health, quality of life and lifestyle changes among pregnant women (4–6).

Previous studies reported the stressful impact in the general population during the early stages of COVID-19 pandemic, and their stressful impact continued to increase over time from January 2020 until April 2020 (1, 12, 13). However, there have been few studies that have reported the mental well-being of pregnant women amidst the early stages of the COVID-19 pandemic (11, 14). During the severe acute respiratory syndrome (SARS) epidemic in 2004, higher rates of death and complications were reported in pregnant women when compared to non-pregnant individuals (15, 16). As the COVID-19 pandemic is still ongoing, there are rising concerns over an increase in stressful impact among pregnant women. Therefore, the aim of the present study was to determine the psychological impact and quality of life among pregnant women amid the early stages of the COVID-19 pandemic.

## Materials and Methods

Quantitative data were collected using a cross-sectional study design which was administered between July 2020 and August 2020 on the Chinese social media platform. Only pregnant of Chinese nationality aged ≥18 years who were Chinese-speaking were eligible for the study. Sampling methods including convenience and snowball samplings were employed in the study. No nmonetary incentives were given to participants for their study participation. The minimum number of participants needed for the study was calculated using the formula as described by Naing et al. (17). The research study protocol was reviewed and approved by the Ethics Committee of the Baoji Maternity and Child Health Care Hospital, and performed in accordance with The Code of Ethics of the World Medical Association (Declaration of Helsinki) and CHERRIES guidelines (18).

### Impact of Event Scale

The self-administered IES questionnaire used in the study has been validated in Chinese language for assessing the extent of psychological impact following the COVID-19 pandemic in Chinese population (11). It is consisted of 15-items and composed of two subscales (i.e., intrusive and avoidance) with a Cronbach's alpha of 0.81. All items in IES questionnaire were scored on a 5-point Likert scale: 0 for not all, 1 for rarely, 3 for sometimes, 5 for often. A total IES score of ≥26 was used to suggest the presence of moderate-to-severe psychological impact following the COVID-19 pandemic.

### Family and Social Support Amid the Early Stages of the COVID-19 Pandemic

Participants were asked to respond to the following questions regarding the impact of the COVID-19 pandemic on family and social support: support from family members, support from friends, sharing of feeling with others, sharing of feeling with other family members, and caring for family members' feeling (with a Cronbach's alpha of 0.84) (1). The response options were based on a 5-point Likert scale. A lower score was used to indicate limited support received from family members and friends following the COVID-19 pandemic (1).

### Mental Health-Related Lifestyle Changes Amid the Early Stages of the COVID-19 Pandemic

Questions regarding the mental health-related lifestyle amid the COVID-19 pandemic including: pay more attention to their mental health, spend more time to relax, rest and exercise were also included in the study. These questions had a Cronbach's alpha of 0.82. The response options were designed on a 5-point Likert scale, ranging from 1 (much decreased) to 5 (much increased) (19, 20). A lower score obtained by participants was used to suggest that there were less favorable changes in their lifestyle amid the COVID-19 pandemic (19, 20).

### Other Measures of Negative Mental Health Impacts Amid the Early Stages of the COVID-19 Pandemic

A self-administered multiple choice questionnaire with a Cronbach's alpha of 0.86 was used to evaluate the negative mental health impacts (e.g., stress from financial, home, work situations) following the COVID-19 pandemic (1). There were six multiple choice questions which were aimed to determine if the participants encountered negative health impacts, including from their workplaces and due to the COVID-19 pandemic based on a 5-point Likert scale ranging from 1 (much decreased) to 5 (much increased) (1).

### Statistical Analysis

Statistical analysis was performed using SPSS ver. 24.0 (IBM Corp., Armonk, NY, USA). Cronbach's alpha was used to measure the reliability and consistency of the factor loadings for the questionnaires used in the study. A Cronbach's alpha value >0.6 was deemed acceptable in social science research (21). Sociodemographic characteristics of participants were evaluated using descriptive statistics. Normally distributed variables were reported as mean ± standard deviation. Categorical variables were presented as frequency [percentage, (%)]. The relationship between independent variables and mental health outcomes was assessed by using Chi-square tests. A *P* < 0.05 was used to denote statistical significance.

## Results

### Participant Characteristics

Of 1,680 participants who were invited to participate in the study, only 1,078 participants were included in the final analysis with a complete rate of 64.2% (Table 1). The reasons for declining to participate were not interested in the study (*n* = 502) and lack of time (*n* = 100). The mean age of participants was 29.4 ± 4.0 years, with more than half of participants (69.9%) had higher qualification. Majority of participants were in 2^nd^ trimester of pregnancy, followed by 3^rd^ and 1^st^ trimesters of pregnancy (27.4 and 23.4%, respectively). More than half of participants were from Southwest China (74.1%), followed by East China (25.2%), Southwest China (0.4%), and South Central China (0.3%). None of the participants were diagnosed positive for the SARS-CoV-2 virus. All participants were married at the time of the study. Only a small minority of participants were healthcare workers (8.0%). More than half of participants were working full-time (57.0%), followed by unemployment (39.1%) and part-time (3.9%).

**Table 1:** Sociodemographic characteristics of pregnant women by trimesters of pregnancy.

	**All** **(***n =*** 1,078)**	**Trimesters**			***P*** **-value**
		**1st** **(***n =*** 252)**	**2nd** **(***n =*** 531)**	**3rd** **(***n =*** 295)**	
Age (years)	29.4 ± 4.0	29.0 ± 3.7	29.6 ± 4.2	29.3 ± 4.1	0.179
**Education level, n (%)**					
Secondary school	325 (30.1)	71 (28.2)	182 (34.3)	72 (24.4)	0.009
Higher qualification	753 (69.9)	181 (71.8)	349 (65.7)	223 (75.6)	
**Healthcare workers, n (%)**					
No	992 (92.0)	18 (7.1)	36 (6.8)	32 (10.8)	0.101
Yes	86 (8.0)	234 (92.8)	495 (93.2)	263 (89.2)	
**Employment status, n (%)**					
Full-time	614 (57.0)	145 (57.5)	298 (56.1)	171 (58.0)	0.074
Part-time	42 (3.9)	17 (6.7)	18 (3.4)	7 (2.4)	
Unemployed	422 (39.1)	90 (35.7)	215 (46.5)	117 (39.7)	

### Family and Social Support Amid the COVID-19 Pandemic

Amid the COVID-19 pandemic, majority of participants reported increased support they received from their family members (91.6%) and friends (78.6%) (Table 2). Also, more than half of participants reported increased sharing of their feeling with their family members (86.9%) and others (92.9%) when they felt blue amid the COVID-19 pandemic. In addition, majority of participants reported increased caring for their family members' (77.4%) amid the COVID-19 pandemic. Participants in 1^st^ and 2^nd^ trimesters of pregnancy were more likely to receive support from family members and care for family members' feelings than participants in 3^rd^ trimester of pregnancy (*P* = 0.020).

**Table 2:** Changes in family and social support by trimesters of pregnancy.

	**Trimesters**			***P*** **-value**
	**1st** **(***n =*** 252)**	**2nd** **(***n =*** 531)**	**3rd** **(***n =*** 295)**	
**Getting support from friends, n (%)**				
Decreased	5 (2.0)	10 (1.9)	2 (0.7)	0.342
Increased	199 (79.0)	423 (79.7)	225 (76.3)	
Same as before	48 (19.0)	98 (18.5)	68 (23.1)	
**Getting support from family members, n (%)**				
Decreased	4 (1.6)	10 (1.9)	2 (0.7)	0.020
Increased	229 (90.9)	496 (93.4)	262 (88.8)	
Same as before	19 (7.5)	25 (4.7)	31 (10.5)	
**Shared feeling with family members, n (%)**				
Decreased	3 (1.2)	10 (1.9)	2 (0.7)	0.668
Increased	221 (87.7)	460 (86.6)	256 (86.8)	
Same as before	28 (11)	61 (11.5)	37 (12.5)	
**Shared feeling with others when feeling blue, n (%)**				
Decreased	4 (1.6)	12 (2.3)	3 (1.0)	0.101
Increased	238 (94.4)	495 (93.2)	268 (90.8)	
Same as before	10 (4.0)	24 (4.5)	24 (8.1)	
**Caring for family members' feelings, n (%)**				
Decreased	1 (0.4)	8 (1.5)	7 (2.4)	0.246
Increased	196 (77.8)	404 (76.1)	234 (79.3)	
Same as before	55 (21.8)	119 (22.4)	54 (18.3)	

### Mental Health-Related Lifestyle Changes Amid the COVID-19 Pandemic

Majority of participants (51.9%) reported that they had paid more attention to their mental well-being and more time to relax (46.1%) amid the COVID-19 pandemic (Table 3). Also, majority of participants reported no changes on the time spent to rest (54.2%). On the other hand, majority of participants reported reduced time spent to exercise (42.0%) amid the COVID-19 pandemic. There were no differences in mental health-related lifestyle among participants with different trimesters of pregnancy (all *P* > 0.05).

**Table 3:** Awareness and lifestyles by trimesters of pregnancy.

	**Trimesters**			***P*** **-value**
	**1st (*n =* 252)**	**2nd (*n =* 531)**	**3rd (*n =* 295)**	
**Pay attention to mental health, n (%)**				
Decreased	6 (2.4)	18 (3.4)	8 (2.7)	0.429
Increased	130 (51.6)	287 (54.0)	142 (48.1)	
Same as before	116 (46.0)	226 (42.6)	145 (49.2)	
**Time spent to rest, n (%)**				
Decreased	9 (3.6)	28 (5.3)	14 (4.7)	0.448
Increased	98 (38.9)	213 (40.1)	132 (44.7)	
Same as before	145 (57.5)	290 (54.6)	149 (50.5)	
**Time spent to relax, n (%)**				
Decreased	77 (30.6)	163 (30.7)	101 (34.2)	0.138
Increased	126 (50.0)	253 (47.6)	118 (40.0)	
Same as before	49 (19.4)	115 (21.7)	76 (25.8)	
**Time spent to exercise, n (%)**				
Decreased	108 (42.9)	205 (38.6)	140 (47.5)	0.086
Increased	96 (38.1)	207 (39.0)	91 (30.8)	
Same as before	48 (19.0)	119 (22.4)	64 (21.7)	

### Attitudes Toward the COVID-19 Pandemic

Majority of participants (54.6%) knew about the SARS-CoV-2 and its relevant prevention knowledge well (Table 4). Also, more than half of participants were concerned about the COVID-19 progress control (57.6%) and thought that COVID-19 pandemic was far away from them (59.2%). More than three-fourth of participants (77.6%) agreed that “pregnant women were more vulnerable to the COVID-19 pandemic than others.” No differences in attitudes toward the COVID-19 pandemic among participants with different trimesters of pregnancy were reported (all *P* > 0.05).

**Table 4:** Attitudes toward COVID-19 by trimesters of pregnancy.

	**Trimesters**			***P*** **-value**
	**1st** **(***n =*** 252)**	**2nd** **(***n =*** 531)**	**3rd** **(***n =*** 295)**	
**Know SARS-CoV-2 and relevant prevention knowledge well, n (%)**				
Yes	131 (52.0)	306 (57.6)	149 (50.5)	0.099
No	121 (48.0)	225 (42.4)	146 (49.5)	
**Concerned about the COVID-19 progress control, n (%)**				
Yes	146 (57.9)	309 (58.2)	166 (56.3)	0.860
No	106 (42.1)	222 (41.8)	129 (43.7)	
**COVID-19 pandemic is far away from me, n (%)**				
Yes	144 (57.1)	318 (59.9)	176 (59.7)	0.752
No	108 (42.9)	213 (40.1)	119 (40.3)	
**Pregnant women are more vulnerable to the COVID-19 than others, n (%)**				
Yes	203 (80.6)	414 (78.0)	220 (74.6)	0.239
No	49 (19.4)	117 (22.0)	75 (25.4)	

### IES

In our study, the overall mean IES in participants was 30.6 ± 12.8, indicating the presence of moderate-to-severe stressful impact amid the COVID-19 pandemic in participants (Table 4). Regardless of their trimesters of pregnancy, all participants had a mean IES score ≥26 (30.9 in 1^st^ trimester of pregnancy, 30.2 in 2^nd^ trimester of pregnancy, and 30.9 in 3^rd^ trimester of pregnancy, P=0.674). Amid the COVID-19 pandemic, more than half of participants who had an IES score ≥26 (63.9%). The overall mean for intrusion and avoidance of participants were 13.9 ± 6.6 and 16.7 ± 7.0, respectively. No differences in mean intrusion and avoidance among participants with different trimesters of pregnancy (all P>0.05).

### Other Measures of Negative Mental Health Impacts Amid the Early Stages of the COVID-19 Pandemic

More than half of participants reported increased work stress (68.5%), financial stress (71.4%) and home stress (59.3%) amid the COVID-19 pandemic (Table 5). In addition, majority of participants reported increased feeling of being horrified (60.8%), apprehensive (61.6%) and helpless (73.7%) amid the COVID-19 pandemic. Only trimester of pregnancy was associated with two of the measures of negative mental health impacts amid the COVID-19 pandemic, which were “increased stress from work” and “increased financial stress” (*P* < 0.05).

**Table 5:** Negative health impacts by trimesters of pregnancy.

	**Trimesters**			***P*** **-value**
	**1st** **(***n =*** 252)**	**2nd** **(***n =*** 531)**	**3rd** **(***n =*** 295)**	
IES	30.9 ± 11.6	30.2 ± 12.8	30.9 ± 13.6	0.674
IES ≥26, n (%)	167 (66.3)	338 (63.7)	184 (62.4)	0.629
**Increased stress from work, n (%)**				
Yes	165 (65.5)	349 (65.7)	224 (75.9)	0.005
No	87 (34.5)	182 (34.3)	71 (24.1)	
**Increased financial stress, n (%)**				
Yes	163 (64.7)	381 (71.8)	226 (76.6)	0.009
No	89 (35.3)	150 (28.2)	69 (23.4)	
**Increased stress from home, n (%)**				
Yes	145 (57.5)	317 (59.7)	177 (60.0)	0.811
No	107 (42.5)	214 (40.3)	118 (40.0)	
**Pregnant women feel horrified due to the COVID-19, n (%)**				
Yes	150 (59.5)	332 (62.5)	173 (58.6)	0.495
No	102 (40.5)	199 (37.5)	122 (41.4)	
**Pregnant women feel apprehensive due to the COVID-19, n (%)**				
Yes	146 (57.9)	334 (62.9)	184 (62.4)	0.390
No	106 (42.1)	197 (37.1)	111 (37.6)	
**Pregnant women feel helpless due to the COVID-19, n (%)**				
Yes	186 (73.8)	383 (72.1)	226 (76.6)	0.374
No	66 (26.2)	148 (27.9)	69 (23.4)	

## Discussion

Our study investigated the psychological impact of the pregnant women in mainland China amid the COVID-19 pandemic during the early stages of the pandemic. In addition, our study provided some nationwide data on the stressful impact and social support in pregnant women. Since the COVID-19 outbreak, an increase of 20% in mental illness cases has been reported in populations (22). However, there are few studies that have investigated the impact of the early stages of COVID-19 pandemic on mental health and quality of life in pregnant women, who have increased risk of developing certain morbidities from COVID-19 because of their physiological changes during pregnancy (11, 23–25). Factors such as inability to purchase food items, fear of becoming sick, and isolation of lockdown may also increase psychological distress, especially among pregnant women with lower socio-demographic status (23). In addition, they have higher risk of suffering poor mental health conditions amid the COVID-19 pandemic (23).

In our study, the overall mean IES was 30.6 ± 12.8 and 63.9% of participants had an IES score ≥26, which provided evidence that our pregnant women experienced moderate-to-severe stressful impact amid the early stages of the COVID-19 pandemic. A study by Wu et al. reported that Chinese pregnant women had higher rate of depressive symptoms amid the COVID-19 pandemic (25). In addition, the authors reported that the COVID-19 pandemic significantly increase the risk of thoughts of self-harm in pregnant women (25). It is suggested that pregnancy is also a factor that can increase vulnerability for developing mental health disorders including anxiety and depression (8).

Our study findings had identified increased stressful impact among pregnant women, which highlighted the strong needs and potential interventions to improve their mental health during this extremely difficult period. Due to the unpredictable nature of the COVID-19 situation, it can trigger some psychological and mental health distresses (1, 13). Several studies reported that exposure to COVID-19 pandemic and its impact on daily life aspects including quality of life, relationship, lifestyle, family and social support are some of the important predictors of the mental health issues (1, 11, 12, 20, 26). Mental health disorders are considered a common cause of morbidity in pregnant women with 22% of women experiencing anxiety and 12% of women experiencing depression (27, 28). It is possible that since pregnant women are more vulnerable to infections during pregnancy due to their naturally suppressed immune system, pregnant women are more likely to be psychologically affected because of the increased morbidity and mortality rate of COVID-19 reported. In addition, they are also further vulnerable to anxiety because of the increased concern about the possible vertical transmission of SARS-CoV-2 to their fetus (25).

Our study reported that overall there was increased family and social support received among pregnant women amid the COVID-19 pandemic. Our results were in line with other studies which reported that amid the COVID-19 pandemic, individuals were more likely to care for their family members' feelings and share their feelings with family members (1, 11, 26). It is suggested that amid the COVID-19 lockdown period, the slower pace of life allows individuals to spend more time supporting and connecting with their family members and friends (1, 26). Therefore, more qualitative and thorough research studies are needed to understand the possible mechanisms behind these findings.

Strengths of our study included the use of validated modified questionnaire to assess psychological impact and quality of life in pregnant women. In addition, our study was one of the first studies to investigate the psychological health impact of the COVID-19 pandemic among pregnant women within the Chinese context. Since our study only involved Chinese pregnant women, our results may not reflect the psychological impact of the larger population of pregnant women from different countries with different severity of COVID-19 pandemic. This is because pregnant women from different countries might experience different severity levels of COVID-19 pandemic and maternal healthcare support provided by the healthcare systems in their countries. On the other hand, pregnant women might more likely to cope with the stress during the COVID-19 pandemic due to the support received from family members and friends, even without the COVID-19 pandemic. Therefore, we recommend more exploratory studies on the mental health status and its associated factors amid the COVID-19 pandemic among pregnant women because these findings are imperative to designing appropriate mental health education for mitigating negative mental health consequences. Our study did not assess the vaccination willingness/status in pregnant women; however, this might have an impact on the quality of life in pregnant women. Therefore, future studies should investigate if there is a difference in quality of life between pregnant women who had and had not been vaccinated against SARS-CoV-2 (29, 30). The association of the support received from family and friends with stress from work, home and financial aspects in pregnant women with different severity of IES scores should be investigated in larger studies. The use of convenience sampling in our participant recruitment may be associated with sampling bias, which limits the generalizability of our findings. In addition, since our results were derived from a cross-sectional study design, it is very challenging and difficult for our study to draw causal inferences. Therefore, our study findings need to be interpreted with caution.

In conclusion, the COVID-19 pandemic has caused several psychological impacts on Chinese pregnant women. Our study provided significant insights regarding the quality of life in pregnant women who remain at high threat for developing mental health problems amidst the pandemic. We recommend that a thoughtful planning and time preparation by the government would definitely help to reduce the negative impacts caused by the COVID-19 pandemic and restore the quality of life among pregnant women.

## Data Availability Statement

The raw data supporting the conclusions of this article will be made available by the authors, upon reasonable request.

## Ethics Statement

The studies involving human participants were reviewed and approved by Ethics Committee of the Baoji Maternity and Child Health Care Hospital. The patients/participants provided their written informed consent to participate in this study.

## Author Contributions

JM, AW, and HZ: conceptualization, formal analysis, methodology, writing—original draft, writing—review, and editing. All authors contributed to the article and approved the submitted version.

## Conflict of Interest

The authors declare that the research was conducted in the absence of any commercial or financial relationships that could be construed as a potential conflict of interest.

## Publisher's Note

All claims expressed in this article are solely those of the authors and do not necessarily represent those of their affiliated organizations, or those of the publisher, the editors and the reviewers. Any product that may be evaluated in this article, or claim that may be made by its manufacturer, is not guaranteed or endorsed by the publisher.

## References

[1] Zhang Y Ma ZF. Impact of the COVID-19 pandemic on mental health and quality of life among local residents in Liaoning Province, China: a cross-sectional study. Int J Environ Res Public Health. (2020) 17:2381. 10.3390/ijerph17072381 32244498 PMC7177660

[2] WHO. Coronavirus Disease (COVID-2019) Situation Reports. (2021). Available online at: https://www.who.int/emergencies/diseases/novel-coronavirus-2019/situation-reports/ (accessed December 2021).

[3] Armitage R Nellums LB. COVID-19 and the consequences of isolating the elderly. Lancet Public health. (2020) 5:e256. 10.1016/S2468-2667(20)30061-X 32199471 PMC7104160

[4] Debolt CA Bianco A Limaye MA Silverstein J Penfield CA Roman AS. Pregnant women with severe or critical coronavirus disease 2019 have increased composite morbidity compared with nonpregnant matched controls. Am J Obstet Gynecol. (2021) 224:510.e511. 10.1016/j.ajog.2020.11.022 33221292 PMC7677036

[5] Zambrano LD Ellington S Strid P Galang RR Oduyebo T Tong VT. Update: characteristics of symptomatic women of reproductive age with laboratory-confirmed SARS-CoV-2 infection by pregnancy status - United States, January 22-October 3, 2020. MMWR Morb Mortal Wkly Rep. (2020) 69:1641–7. 10.15585/mmwr.mm6944e3 33151921 PMC7643892

[6] Villar J Ariff S Gunier RB Thiruvengadam R Rauch S Kholin A. Maternal and neonatal morbidity and mortality among pregnant women with and without COVID-19 infection: The INTERCOVID Multinational Cohort Study. JAMA Pediatr. (2021) 175:817–26. 10.1001/jamapediatrics.2021.1050 33885740 PMC8063132

[7] Banerjee D. The impact of COVID-19 pandemic on elderly mental health. Int J Geriatr Psychiatry. (2020) 35:1466–7. 10.1002/gps.5320 32364283 PMC7267435

[8] López-Morales H Del Valle MV Canet-Juric L Andrés ML Galli JI Poó F. Mental health of pregnant women during the COVID-19 pandemic: A longitudinal study. Psychiatry Res. (2021) 295:113567. 10.1016/j.psychres.2020.113567 33213933 PMC7657008

[9] Loomans EM Van Dijk AE Vrijkotte TG Van Eijsden M Stronks K Gemke RJ. Psychosocial stress during pregnancy is related to adverse birth outcomes: results from a large multi-ethnic community-based birth cohort. Eur J Public Health. (2013) 23:485–91. 10.1093/eurpub/cks097 22850187

[10] Stein A Pearson RM Goodman SH Rapa E Rahman A Mccallum M. Effects of perinatal mental disorders on the fetus and child. Lancet. (2014) 384:1800–19. 10.1016/S0140-6736(14)61277-0 25455250

[11] Zhang Y Ma ZF. Psychological responses and lifestyle changes among pregnant women with respect to the early stages of COVID-19 pandemic. Int J Soc Psychiatry. (2020) 67:344–50. 10.1177/0020764020952116 32815434 PMC8191160

[12] Qiu J Shen B Zhao M Wang Z Xie B Xu Y. A nationwide survey of psychological distress among Chinese people in the COVID-19 epidemic: implications and policy recommendations. Gen Psychiatry. (2020) 33:e100213. 10.1136/gpsych-2020-100213 32215365 PMC7061893

[13] Zhang Y Wen C Zhang Y Luo X Ma ZF. The impact of mental health and stress concerns on relationship and sexuality amidst the COVID-19 lockdown. J Sexual Med. (2021) 18:1843–50. 10.1016/j.jsxm.2021.06.013 34535368 PMC8569526

[14] Dong H Hu R Lu C Huang D Cui D Huang G. Investigation on the mental health status of pregnant women in China during the Pandemic of COVID-19. Arch Gynecol Obstet. (2021) 303:463–9. 10.1007/s00404-020-05805-x 33009997 PMC7532741

[15] Lam CM Wong SF Leung TN Chow KM Yu WC Wong TY. A case-controlled study comparing clinical course and outcomes of pregnant and non-pregnant women with severe acute respiratory syndrome. BJOG. (2004) 111:771–4. 10.1111/j.1471-0528.2004.00199.x 15270922 PMC7161819

[16] Wong SF Chow KM Leung TN Ng WF Ng TK Shek CC. Pregnancy and perinatal outcomes of women with severe acute respiratory syndrome. Am J Obstet Gynecol. (2004) 191:292–7. 10.1016/j.ajog.2003.11.019 15295381 PMC7137614

[17] Naing L Winn T Rusli BN. Practical issues in calculating the sample size for prevalence studies. Arch Orofac Sci. (2006) 1:9–14.8804140

[18] Eysenbach G. Improving the quality of Web surveys: the Checklist for Reporting Results of Internet E-Surveys (CHERRIES). J Med Internet Res. (2004) 6:e34. 10.2196/jmir.6.3.e34 15471760 PMC1550605

[19] Lau JT Yang X Tsui HY Pang E Wing YK. Positive mental health-related impacts of the SARS epidemic on the general public in Hong Kong and their associations with other negative impacts. J Infect. (2006) 53:114–24. 10.1016/j.jinf.2005.10.019 16343636 PMC7132442

[20] Cao Y Ma ZF Zhang Y Zhang Y. Evaluation of lifestyle, attitude and stressful impact amid COVID-19 among adults in Shanghai, China. Int J Environ Health Res. (2020) 9:1–10. 10.1080/09603123.2020.1841887 33164565

[21] Shammi M Bodrud-Doza M Towfiqul Islam ARM Rahman MM. COVID-19 pandemic, socioeconomic crisis and human stress in resource-limited settings: A case from Bangladesh. Heliyon. (2020) 6:e04063. 10.1016/j.heliyon.2020.e04063 32462098 PMC7242967

[22] Banna MHA Sayeed A Kundu S Christopher E Hasan MT Begum MR. The impact of the COVID-19 pandemic on the mental health of the adult population in Bangladesh: a nationwide cross-sectional study. Int J Environ Res Public Health. (2020) 2:1–2. 10.1080/09603123.2020.1802409 32741205

[23] Berthelot N Lemieux R Garon-Bissonnette J Drouin-Maziade C Martel É Maziade M. Uptrend in distress and psychiatric symptomatology in pregnant women during the coronavirus disease 2019 pandemic. Acta Obstet Gynecol Scand. (2020) 99:848–55. 10.1111/aogs.13925 32449178

[24] Saccone G Florio A Aiello F Venturella R De Angelis MC Locci M. Psychological impact of coronavirus disease 2019 in pregnant women. Am J Obstet Gynecol. (2020) 223:293–5. 10.1016/j.ajog.2020.05.003 32387321 PMC7204688

[25] Wu Y Zhang C Liu H Duan C Li C Fan J. Perinatal depressive and anxiety symptoms of pregnant women during the coronavirus disease 2019 outbreak in China. Am J Obstet Gynecol. (2020) 223:240.e241-240.e249. 10.1016/j.ajog.2020.05.009 32437665 PMC7211756

[26] El-Zoghby SM Soltan EM Salama HM. Impact of the COVID-19 pandemic on mental health and social support among adult Egyptians. J Comm Health. (2020) 45:689–95. 10.1007/s10900-020-00853-5 32468155 PMC7255077

[27] Palladino CL Singh V Campbell J Flynn H Gold KJ. Homicide and suicide during the perinatal period: findings from the National Violent Death Reporting System. Obstet Gynecol. (2011) 118:1056–63. 10.1097/AOG.0b013e31823294da 22015873 PMC3428236

[28] Woody CA Ferrari AJ Siskind DJ Whiteford HA Harris MG. A systematic review and meta-regression of the prevalence and incidence of perinatal depression. J Affect Disord. (2017) 219:86–92. 10.1016/j.jad.2017.05.003 28531848

[29] Zhang Y Luo X Ma ZF. Willingness of the general population to accept and pay for COVID-19 vaccination during the early stages of COVID-19 pandemic: a nationally representative survey in mainland China. Hum Vaccin Immunother. (2020) 17:1622–7. 10.1080/21645515.2020.1847585 33606600 PMC8115554

[30] Liu D Luo L Xie F Yu Z Ma ZF Wang Y. Factors associated with the willingness and acceptance of SARS-CoV-2 vaccine from adult subjects in China. Hum Vaccin Immunother. (2021) 17:2405–14. 10.1080/21645515.2021.1899732 33759691 PMC8475565

